# Characteristics of upright versus supine reflux pattern in patients with laryngopharyngeal reflux

**DOI:** 10.1016/j.bjorl.2019.08.003

**Published:** 2019-09-18

**Authors:** Tamer A. Mesallam, Abdulsalam A. Baqays

**Affiliations:** a King Saud University, Department of Otolaryngology, Head and Neck Surgery, Riyadh, Saudi Arabia; b King Saud University, Otolaryngology Department, Research Chair of Voice, Communication, and Swallowing Disorders, Riyadh, Saudi Arabia

**Keywords:** LPR patterns, Upright LPR, Supine LPR, Oro-pharyngeal pH monitoring

## Abstract

**Introduction:**

Many laryngeal-related problems have been attributed to laryngopharyngeal reflux including dysphonia, frequent throat clearing, chronic cough, and globus sensation. However, there is still controversy regarding diagnosis and clinical presentation of this disorder.

**Objective:**

The main objective of this study is to describe laryngopharyngeal reflux characteristics of different reflux position patterns in laryngopharyngeal reflux patients diagnosed with oropharyngeal pH monitoring.

**Methods:**

A retrospective chart review was conducted for 161 laryngopharyngeal reflux patients diagnosed with 24 h oro-pharyngeal pH monitoring. Study subjects were categorized into upright and supine laryngopharyngeal reflux groups based on the pH results. The two groups were compared regarding the clinical presentation and pH characteristics.

**Results:**

Significant higher rates of upright laryngopharyngeal reflux position than supine laryngopharyngeal reflux position (P < 0.0001) were reported among the study group. Reflux symptoms index results were significantly higher in the upright larybgopharyngeal reflux group compared to the supine laryngopharyngeal reflux group. 24 h oropharyngeal pH measurements composite Ryan score was significantly higher in the upright group compared to the supine group (P < 0.0001). No significant difference was found between the upright and supine laryngopharyngeal reflux groups regarding the frequency of clinical presentation or voice handicap index ratings.

**Conclusion:**

Laryngopharyngeal reflux was found to be more prevalent occurring in the upright position among the study group. Reflux-related characteristics including pH parameters were more evident in the upright laryngopharyngeal reflux position.

## Introduction

Laryngopharyngeal reflux (LPR) is defined as upward movement of gastric contents up to the laryngopharyngeal area.^1^ Many laryngeal disorders have been attributed to LPR including; reflux laryngitis, subglottic stenosis, contact ulcers and granulomas, and even laryngeal carcinoma.^2^, ^3^, ^4^, ^5^ However, a lot of controversy is found regarding the clinical presentation and diagnosis of LPR. Dysphonia, frequent throat clearing, globus sensation, chronic cough, postnasal discharge, and dysphagia have been mentioned in many literatures as symptoms related to LPR. However, there is also a lot of controversy regarding the association between these symptoms and LPR.^6^, ^7^, ^8^

The oro-pharyngeal Dx-pH measurement system is a known diagnostic technique for LPR that has been reported to be sensitive in detecting acidic laryngopharyngeal reflux events. It uses a thin trans-nasal probe that can be easily advanced through the nose to the oropharynx. The tip of the probe has a sensor that can detect liquid and aerosolized forms of acid. The sensor records pH values twice every second and monitors hydration that can eliminate data if the tip dries out to avoid pseudo-reflux events.^9^, ^10^

Unlike gastroesophageal reflux (GER), LPR has been described widely among otolaryngology practice. One of the main differences that has been reported between LPR and GER is the pattern of occurrence of reflux episodes. Reflux episodes in LPR usually occur in the upright position during the daytime, while reflux episodes in GER occur more often in the supine position at nighttime or during sleep.^11^, ^12^ Recently, a study by Scott et al.^13^ reported that LPR occurred exclusively in the supine position. In another earlier study by Beaver et al.^14^ LPR was found to be more frequently reported in the supine position than upright position. Interestingly the above two studies used the recent technology of 24 h oropharyngeal pH monitoring in diagnosing patients with LPR.

Given the controversy regarding describing the pattern of laryngopharyngeal reflux events in terms of being primarily occurring in the upright or the supine position. The value of this study is to explore the position pattern of laryngopharyngeal reflux events being measured by oro-pharyngeal pH monitoring and to compare the reflux characteristics in each pattern

## Subjects and methods

The Institutional Review Board of the College of Medicine at King Saud University has approved the study under IRB #E-16-1928. A retrospective chart review was carried out for all patients who underwent 24 h oro-pharyngeal pH monitoring between January 2014 and July 2015. All adult patients with completed 24 h oro-pharyngeal pH monitoring during that period were included in the study. According to the pH monitoring study protocol at our center, all patients had to stop any anti-LPR medications 2 weeks before the date of study. Thus all patients underwent the study with less than 2 weeks off-medications were excluded. Also patients with incomplete data of voice handicap index-10 (VHI-10) and reflux symptom index (RSI) were excluded from the study.

LPR was assessed using 24 h oro-pharyngeal pH testing and according to the results, study subjects were categorized into two groups. Patients with positive acidic reflux results in the upright position were included in the upright LPR group while those with positive acidic reflux results in the supine position were included in the supine LPR group. The two groups were compared regarding clinical presentation, results of the composite parameters of the 24 h oro-pharyngeal pH monitoring as well as the results of VHI-10 and RSI.

### 24-hour oro-pharyngeal pH monitoring

24 h oro-pharyngeal Dx-pH probe system was used to confirm the diagnosis og LPR. After probe insertion, patients were given instructions regarding recording the meal and supine position time in the device and on an external diary form while keeping their normal daily activities as usual during the study.

Following 24 h of recording, the data was analyzed using the system’s software. Thresholds for identifying acidic reflux events were set 5.5 and 5 for the upright and supine positions, respectively. After excluding the meal times, the system automatically generates pH measurements based on the pH thresholds for both upright and supine positions. Three main parameters are incorporated in these measures including number of reflux episodes, the duration of longest reflux episode, and the percentage of time below the predetermined pH threshold. Also the system generates a total composite score called Ryan Score based on the 3 above-mentioned parameters. Scores greater than 9.41 in the upright position and 6.80 in the supine position were considered suggestive of LPR.^15^

### Statistical analysis

Based on the normality test, non-parametric statistics were used to compare between variables in this study. The Mann–Whitney test was used to examine differences between groups and Chi-square test was used to compare qualitative variables with a level of significance at 0.05. The Statistical Package for the Social Sciences, version 17 (SPSS Inc., Chicago, Ill., USA) was used for all statistical analysis.

## Results

### Patients’ demographics

The study included 161 patients suffering from LPR-related presentation and underwent 24 h oro-pharyngeal pH monitoring. There were 97 female and 64 male subjects participated in the study with a mean age (±SD) of 45.31(±13.92) years old.

### 24-hour oro-pharyngeal pH results

Table 1 shows the distribution of the LPR pattern among the study group. The results of oro-pharyngeal pH monitoring showed that out of the 161 patients there were 82 patients with non-acidic reflux results while 79 patients had acidic reflux. Among patients with detected acidic reflux, 62% had acidic reflux in the upright position, 25% had acidic reflux in the supine position, and 13% had acidic reflux in both supine and upright positions. There were significantly higher numbers of patients with upright LPR compared to supine LPR. Moreover, on comparing the upright LPR group with the supine LPR group regarding the pH results, there were significantly higher numbers of LPR episodes and total Ryan score in the upright group compared to the supine group. However, the percent of time below base-line pH and the longest reflux episodes parameters were significantly higher in the supine group (Table 2).

**Table 1:** Distribution of laryngopharyneal reflux results among the study group.

Oropharyngeal pH results	n (%)	Pattern
Non detected acidic LPR	82 (51%)	
Detected Acidic LPR	79 (49%)	Upright acidic LPR 49 (62%)
		Supine acidic LPR 20 (25%)
		Mixed upright and supine LPR 10 (13%)
Total	161

**Table 2:** Comparison between upright and supine LPR regarding oropharyngeal pH monitoring results

	Upright LPR	Supine LPR	P
Number of patients n (%)	49 (62%)	20 (25%)	<0.0001
Number of acidic reflux episodes $\bar{X}$ (SD)	18.18 (17.08)	11.55 (6.17)	0.02
% of time below base line pH $\bar{X}$ (SD)	1.44 (1.98)	3.48 (2.81)	0.006
Longest reflux episode $\bar{X}$ (SD)	4.49 (4.79)	10.16 (6.19)	0.001
Ryan Score $\bar{X}$ (SD)	58.65 (63.69)	16.16 (12.09)	< 0.0001

### VHI-10 and RSI results

Table 3 shows the comparison between the upright LPR group and the supine LPR group regarding VHI-10 and RSI ratings. RSI scores were significantly higher in the upright LPR group compered to the supine group. On the other hand, and despite having higher VHI-10 scores in the upright LPR group, the difference did not reach significant levels between the two groups.

**Table 3:** Comparison between upright and supine LPR regarding VHI and RSI results

	Upright LPR $\bar{X}$ (SD)	Supine LPR $\bar{X}$ (SD)	P
VHI score	11.57 (11.31)	8.02 (11.32)	0.26
RSI score	19.97 (10.22)	12.3 (5.76)	<0.0001

### Clinical presentation results

Non-specific laryngoscopic findings were reported among the study group including erythema of the posterior larynx (70%), subglottic edema or pseudo sulcus (66%), arytenoid edema, and inter-arytenoid hypertrophy (61%). Dysphonia, frequent throat clearing, globus sensation, and cough were the clinical presentations investigated among the two groups in this study. Interestingly, none of the above-mentioned symptoms showed significant difference on comparing the clinical presentation of the two groups (Table 4)

**Table 4:** Comparison between upright and supine LPR regarding distribution of clinical presentation

	Upright LPR (n = 49) n (%)	Supine LPR (n = 20) n (%)	P
Dysphonia	20 (29%)	17 (25%)	0.62
Globus sensation	9 (13%)	8 (12%)	0.80
Frequent throat clearing	9 (13%)	5 (7%)	0.28
Cough	14 (20%)	7(10%)	0.12

## Discussion

The main aim of this study was to explore the reflux-related characteristics of position patterns among a group of laryngopharyngeal reflux patients diagnosed with 24 h oro-pharyngeal pH monitoring. Laryngopharyngeal reflux can be classified based on content of the reflux into acidic and non-acidic LPR. The present study reveals that half of the study group found to have acidic reflux. This group was further divided based on positional pattern of occurrence into upright reflux, supine reflux, and dual positional reflux. Indeed, Upright acidic reflux group were the predominant group while the minority was accounted to dual positional and supine group. This finding was matching the results of Ayazi et al.^15^ and Koufman et al.^16^ studies. Hoppo et al.^17^ explained the pathophysiological mechanism of LPR in relation to body's positions. They reported that patients with upright LPR have competent anti-reflux barriers while they are in supine position. Once they are shifting to upright position intra-gastric air will come in contact with lower esophageal sphincter (LES) accompanying the increase of intra-abdominal pressure. Thus, LES will relax in response to stimulation of gastric stretch receptors and upright LPR will occur. Additionally, detailed analysis of the 24 h oro-pharyngeal pH measurements in the current study including numbers of acidic reflux episode, time percent below pH baseline, and Ryan score revealed significantly higher number of reflux episodes in the upright group compared to the supine group. Moreover, despite having significantly higher rates of longest reflux episode and percent of time below base-line pH in the supine group which may be due to the delay in acid clearance related to the supine position, the total composite Ryan score of the pH study was significantly higher in the upright group. These findings confirm the hypothesis of having laryngopharyngeal reflux episodes occurring mainly in the upright position. Although using the same diagnostic technology for LPR, these findings contradict with the findings of the previously mentioned studies of Scott et al.^13^ and Beaver et al.^14^

In the present study, we used a 24 h oro-pharyngeal Dx-pH probe system (Restech Corp., San Diego, CA) for diagnosing LPR. This technology has been proved to be sensitive in detecting acidic laryngoparyngeal reflux episodes compared to traditional dual pH probe techniques. In their study, Golub et al.^9^ reported that the results of oropharyngeal pH correlate closely to that of the proximal probe results of the dual pH studies when measuring acid exposure duration. Also, they found this technology to be more sensitive than dual pH monitoring in detecting pH fluctuation. Moreover, oropharyngeal pH has been reported by Yuksel et al.^18^ to be more sensitive than traditional pH monitoring in evaluation of patients with extraesophageal reflux. On the other hand, the results of the oropharyngeal pH in comparison to muti-channel intramural impedance pH (MII-pH) is controversial. Most of the studies reported variable proportions between oropharyngeal pH events and preceded gastroesophageal event detected by MII-pH ranging from 17% to 60%.^19^, ^20^, ^21^ However, one possible explanation for this variable correlation between the two techniques is that not all the gastroesophageal reflux episodes can reach up to the laryngopharyngeal area. These results support the reliability of oropharyngeal pH technique in addition of being less invasive procedure compared to other pH techniques.^9^

A report from Johnston et al. studied GERD and presented that pH cutoff above 4 will lead to cellular injury of laryngeal mucosa.^22^ Moreover, Wiener et al. presented that a median of 4 in the distal esophagus is equivalent to approximately 5.5 in laryngopharynx.^23^ In the current study, we used pH threshold of 5.5 and 5 to diagnose positive reflux in upright, and supine position respectively as reported in Ayazi et al.^15^ In our study cohort, number of patients with upright LPR was significantly higher than supine LPR. This was in accordance with other published results in previous studies.^15^, ^24^

The reflux symptom index (RSI) is one of the non-instrumental tests used to detect the possibility of LPR that was developed in 2002. RSI is a validated scoring system, which possess 9 questions regarding presence of symptoms that suggestive of LPR.^25^ RSI have been translated and validated into several languages. Farahat et al. published an Arabic validation of the RSI that maintains the same validity as the original one.^26^ In our study, RSI scores were significantly higher in the upright LPR group compared to the supine one. These findings add a confirmatory support to general notion of predominant upright pattern of LPR over the supine position.

Voice Handicap Index-10 (VHI-10) is an outcome measurement which was developed by Rosen et al.^27^ for detecting self-perception of voice problems. This scale has 3 main domains that are physical, functional, and emotional domains. In the interpretation of this scale, Severity of voice problem is proportionally related to higher score in the scale. Furthermore, this scale has been validated to be used in Arabic as many other languages. In this study, we used the Arabic Voice Handicap index-10 (VHI-10)^28^ in order to elicit inferential information between both groups. Interestingly, no-significant difference was found between the upright and supine LPR groups. Similarly, laryngeal symptoms reported by patients have been studies between the two study groups. Dysphonia was cited as a common complaint amongst both groups. However, the least cited complains were globus sensation and frequent throat cleaning for upright group. While the least cited in the supine group was frequent throat cleaning. In the current study, no significant difference was found between the two groups regarding clinical presentation. This could be due to that most of the symptoms reported by patients in this study such as dysphonia, throat clearing, globus, and cough are nonspecific to LPR and could be a common presentation for other problems including allergy, post-nasal drip, and voice misuse.^6^, ^7^, ^8^, ^17^, ^29^

## Conclusion

The results of this study showed that laryngopharyngeal reflux can occur either in the upright or supine position but it is more commonly reported in the upright position regardless the technology used for pH monitoring. Moreover, RSI rating and pH composite score were found to be higher in the upright position compared to supine position. It seems that most of the voice-related clinical presentations including dysphonia, are non-specific to laryngopharyngeal reflux.

## Conflicts of interest

The authors declare no conflicts of interest.
